# Insights into the tumor microenvironment of B cell lymphoma

**DOI:** 10.1186/s13046-022-02579-9

**Published:** 2022-12-29

**Authors:** Wern Lynn Ng, Stephen M. Ansell, Patrizia Mondello

**Affiliations:** Division of Hematology, Mayo Clinic, 200 1st St SW, Rochester, MN 55905 USA

**Keywords:** Tumor microenvironment, B-cell lymphoma, T cells, T follicular helper cells, T regulatory cells, Tumor-associated macrophages, Myeloid-derived suppressor cells, Cancer-associated fibroblasts

## Abstract

The standard therapies in lymphoma have predominantly focused on targeting tumor cells with less of a focus on the tumor microenvironment (TME), which plays a critical role in favoring tumor growth and survival. Such an approach may result in increasingly refractory disease with progressively reduced responses to subsequent treatments. To overcome this hurdle, targeting the TME has emerged as a new therapeutic strategy. The TME consists of T and B lymphocytes, tumor-associated macrophages (TAMs), myeloid-derived suppressor cells (MDSCs), cancer-associated fibroblasts (CAFs), and other components. Understanding the TME can lead to a comprehensive approach to managing lymphoma, resulting in therapeutic strategies that target not only cancer cells, but also the supportive environment and thereby ultimately improve survival of lymphoma patients. Here, we review the normal function of different components of the TME, the impact of their aberrant behavior in B cell lymphoma and the current TME-direct therapeutic avenues.

## Introduction

The last two decades have seen numerous discoveries which have helped understand the biology of B cell lymphoma and lay the foundation for precision therapies. B cell lymphomas arise from the germinal center (GC), a dynamic structure that forms upon encounter of naïve B cells with a putative antigen [1], and may be secondary to i) genetic/epigenetic alterations in the GC B cells or ii) aberrant response of immune components of the microenvironment ultimately leading to lymphomagenesis [2]. Gene expression profiling (GEP) studies have divided diffuse large B cell lymphoma (DLBCL) - the most common B cell lymphoma - into two main subgroups based on the cell of origin (COO): the activated B cell (ABC) and the germinal center B cell (GCB) subtypes [3]. More recently, two additional molecular classifications have used whole exome sequencing (WES) and structural genomic abnormalities to further subdivide DLBCL into several genetically defined subgroups [4, 5]. An additional layer of complexity includes the immune cells that infiltrate the tumor. A landmark study performed on tumor biopsies from 95 untreated patients with follicular lymphoma (FL) - the second most frequent B cell lymphoma - demonstrated significant enrichment of genes associated with macrophages in patients with unfavorable outcomes whereas the expression signature was enriched for genes linked to T-cells in those with a favorable outcome [6]. Additionally, we reported the prognostic value of memory CD4+ T-cells, which play a critical role in immune surveillance, and designed a prognostic risk model (BioFLIPI) to improve the identification of high-risk patients [7]. Similarly, the prognostic relevance of TME in DLBCL has been uncovered in two recent studies which have further deconvoluted the TME in several ecosystems [8, 9]. Part of the reason for an unfavorable TME may be linked to the mutation of genes directly or indirectly involved in the control of antigen presentation, including CREBBP [10], EP300 [11], EZH2 [12], and others [13]. However, many additional mechanisms may come into play to shape the immune response against tumors [14]. Here, we dissect the function of different immune components of the TME (Table 1), the impact of their aberrant expression in B cell lymphoma and novel therapeutic avenues (Tables 2 and 3).

**Table 1:** Role and markers of the immune cells of the TME

Immune cells	Role	Markers
Tfh cells	- formation and maintenance of GCs - promote clonal selection and affinity maturation of GC B cells	CD4^+^, CXCR5^+^, PD1^+^, ICOS^+^
Treg cells	- prevent autoimmunity by suppressing immune response activation and promoting tolerance towards self-antigens - suppress tumor immunity leading to immune escape	CD4^+^, CD25^+^, FoxP3^+^, CD127^-^
Effector CD8+ T cells	Highly cytotoxic against transformed and virus-infected cells	CD8^+^, CD45RA^+^, CD45RO^-^, CCR7^+^, CD28^+^, IFN- *γ*^+^, IL-2^+^
TAMs	M1 – anti-tumorigenic M2 – pro-tumorigenic: i) suppress antitumor immunity by inhibiting the recruitment and activation of T cells; ii) serve as metastasis promoters	M1: CD80^+^, CD86^+^, CD64^+^, CD16^+^, CD32^+^ M2: CD163^+^, CD206^+^, CD204^+^
MDSCs	- enhance tumorigenesis by enhancing migratory capacity, autocrine growth factor-induced signaling and increasing levels of secretory molecules	HLA-DR^-^, CD14^+^, CD11b^+^, CD33^+^, S100A9^+^,
CAFs	- enhance stiffening of ECM, angiogenesis, and cancer cell invasion - promote an immune suppressive TME	PDGFRA^+^, PDGFRB^+^, FSP-1/S100A4^+^ and FAP^+^
NK cells	- prevention of infection and tumor growth	CD56^dim^ CD16^bright^ (90%) CD56^bright^ CD16^dim^ (10%)
ILCs	- regulate tissue homeostasis, inflammation, tumor surveillance and tumorigenesis	CD45^+^, CD127^+^, CD161^+/-^, HLA-DR^+^, CD56^+/-^, CD11b^-^, CD11c^+/-^, CD19^-^

*Abbreviations:* *Tfh* T follicular helper, Treg T regulatory, TAMs Tumor-associated macrophages, *MDSCs* Myeloid-derived suppressor cells, *CAFs* cancer-associated fibroblasts, *ECM* extracellular matrix, *TME* tumor microenvironment, *PDGFRA* platelet derived growth factor receptor *α*, *PDGFRB* platelet derived growth factor receptor *β*, *FSP* fibroblast specific protein 1, *FAP* fibroblast activation protein, *NK* Natural Killer, *ILCs* Innate lymphoid cells

**Table 2:** Clinical trials including agents targeting the immune cells of the TME in B cell lymphomas

Immune cells	Identifier	Study	Phase	Target	Agent
Tfh cells	NCT02376699	A Phase 1, Open-label, Dose-escalation Study of SEA-CD40 in Adult Patients with Advanced Malignancies	I	CD40	SEA-CD40
Treg cells	NCT04855253	Phase I/II Trial Using E7777 to Enhance Regulatory T-cell Depletion Prior to Tisagenlecleucel (Kymriah) Therapy for Relapsed/Refractory DLBCL	I/II	IL-2	E7777
	NCT01919619	A Pilot Study of Lenalidomide Alternating with Ipilimumab Post Allogeneic and Autologous Stem Cell Transplantation	II	CTLA-4	Ipilimumab
	NCT04544059	Lenalidomide Plus R-CHOP for CNS Relapse Prophylaxis in DLBCL	II	CD28	Lenalidomide
	NCT05429	A Phase 3, Single-Arm, Open-Label, Multicenter Study to Evaluate the Safety and Efficacy of Tafasitamab Plus Lenalidomide in Participants with Relapsed or Refractory DLBCL	III	CD28	Lenalidomide
	NCT04432402	Efficacy and Safety of Lenalidomide in Combination with R-GemOx in First-line treatment of Elderly DLBCL	N/A	CD28	Lenalidomide
	NCT04432402	Duvelisib Exposure to Enhance Immune Profiles of T cells in Patients with DLBCL (DEEP T CELLS)	I	PI3K	Duvelisib
	NCT04849351	A Multi-center, Single-arm, Open-label Clinical Study to Evaluate the Efficacy and Safety of HMPL-689 in Patients with Relapsed/Refractory MZL and FL	II	PI3K	Amdizalisib, HMPL-689
	NCT03314922	A Phase 1b, Open-Label, Dose-Escalation Study for the Safety, Tolerability, and Pharmacokinetics of INCB050465 in Japanese Subjects with Previously Treated B-cell lymphoma (CITADEL-111)	I	PI3K	Parsaclisib
	NCT03919175	A Phase 2 Study of Umbralisib and Rituximab as Initial Therapy for Patients with FL and MZL	II	PI3K	Umbralisib
	NCT02367040	A Phase III, Randomized, Double-blind, Placebo-controlled Study Evaluating the Efficacy and Safety of Copanlisib in Combination with Rituximab in Patients with Relapsed iNHL – CHRONOS-3	III	PI3K	Copanlisib (Aliqopa, BAY80-6946)
	NCT03884998	A Phase 1 study of PI3Kα,δ Inhibitor Copanlisib in Combination with PD-1 Antagonist Nivolumab in Patients with Transformed CLL (Richter’s Transformation) or NHL	I	PI3K	Copanlisib (Aliqopa, BAY80-6946)
Effector CD8+ T cells	NCT04566978	A Pilot Study of 89Zr-DFO-REGN3767 Anti LAG-3 Antibody Positron Emission Tomography in Patients with Relapsed/Refractory DLBCL	I	LAG3	89Zr-DFO-REGN3767
	NCT05039658	A Phase Ib, Open Label, Randomized, Multicenter Study of the Efficacy and Safety of IBI110 Single Agent and in Combination with Sintilimab for Patients with Relapsed or Refractory DLBCL	Ib	LAG3	IBI110
	NCT02061761	A Phase 1/2a Dose Escalation and Cohort Expansion Study of the Safety, Tolerability, and Efficacy of Anti-LAG-3 Monoclonal Antibody (Relatlimab, BMS-968016) Administered Alone and in Combination with Anti-PD-1 Monoclonal Antibody (Nivolumab, BMS-936558) In relapsed or Refractory B-cell Malignancies	I/IIA	LAG3	Relatlimab, BMS-896016
	NCT05255601	A Phase I/II study of the Safety, Tolerability, Pharmacokinetics and Preliminary Efficacy of Relatlimab Plus Nivolumab in Pediatric and Young Adult Participants with Recurrent or Refractory Classical HL and NHL	I/II	LAG3	Relatlimab, BMS-896016
	NCT04767308	A Single-center, Single-arm Exploratory Clinical Trial to Evaluate the Safety and Efficacy of Fully Human Anti-CD5 Chimeric Antigen Receptor T cells (CT125A Cells) for the Treatment of Relapsed/Refractory CD5+ Hematopoietic Malignancies	I	CD5	CD125A cells
	NCT01919619	A Pilot Study of Lenalidomide Alternating with Ipilimumab Post Allogeneic and Autologous Stem Cell Transplantation	II	CTLA4	Ipilimumab
TAMs	NCT03530683	A Phase1a/1b Dose-Escalation and Expansion Trial of TTI-622 in patients with Advanced Hematologic Malignancies, Including Lymphoma, Leukemia, and Multiple Myeloma	I	SIRPα	TTI-622 (SIRPα-IgG4 Fc)
	NCT05507541	Randomized Phase 2 Study with Safety Run-In of PD-1 Inhibitor and IgG4 SIRPα-Fc Fusion Protein (TTI-622) and PD-1 Inhibitor and IgG1 SIRPα-Fc Fusion Protein (TTI-621) in Relapsed DLBCL	II	SIRPα	TTI-622 (SIRPα-IgG4 Fc)
	NCT02953509	A Phase 1b/2 Trial of Hu5F9-G4 in Combination with Rituximab or Rituximab + Chemotherapy in Patients with Relapsed/Refractory B-cell NHL	Ib/II	CD47	Hu5F9-G4
	NCT05626322	A Phase 1b/2 Study of PF-07901801, a CD47 Blocking Agent, with Tafasitamab and Lenalidomide for Participants with Relapsed/Refractory DLBCL Not Eligible for Stem Cell Transplantation	II	CD47	PF-07901801
	NCT05025800	A Phase I/II Open Label, Single Center, Study of the Combination of ALX148, Rituximab and Lenalidomide in Patients with Indolent and Aggressive B-cell NHL	I/II	CD47	ALX148
	NCT04806035	A Phase 1b Multi-cohort Study of TG-1801 Alone in Combination with Ublituximab in Subjects with B-cell Lymphoma or CLL	I	CD47	TG-1801
MDSCs	NCT03711604	An Open Label, Compassionate Use Study of Tenalisib (RP6530) in Patients Currently Receiving Treatment on Tenalisib Trials in Hematological Malignancies	I/II	PI3K δ/γ	Tenalisib (RP6530)
	NCT02916979	A Pilot Trial Examining Myeloid-Derived Suppressor Cells and Checkpoint Immune Regulators’ Expression in Allogeneic Stem cell Transplant Recipients Using Myeloablative Busulfan and Fludarabine	I		Myeloablative Busulfan and Fludarabine
CAFs	NCT03155620	NCI-COG Pediatric MATCH (Molecular Analysis for Therapy Choice Screening Protocol)	II	FGFR	JNJ-42756493
	NCT02465060	Molecular Analysis for Therapy Choice (MATCH)	II	FGFR	JNJ-42756493
NK cells	NCT03056339	Dose Escalation Study Phase I/II of Umbilical Cord Blood-Derived CAR-Engineered NK Cells in Conjunction with Lymphodepleting Chemotherapy in Patients with Relapsed/Refractory B-Lymphoid Malignancies	I/II	CD19	iC9/CAR.19/IL15-Transfuced CB-NK Cells
	NCT04052061	Open-Label, Phase I Study of CD19 t-haNK in Subjects with DLBCL who have Received 2 or More Lines of Therapy and Are Ineligible for Transplant	I	CD19	CD19 t-haNK
	NCT04074746	Bispecific NK Engager AFM13 Combined with NK Cells for Patients with Recurrent of Refractory CD30 Positive HL or NHL	I/II	CD30	AFM13
	NCT02890758	Phase I Trial of Universal Donor NK Cell Therapy in Combination with ALT-803	I	IL-15	ALT-803
	NCT04609579	A Phase 1 Open-label of Study SNX281 Given as Monotherapy and in Combination with a Checkpoint Inhibitor in Subjects with Advanced Solid Tumors and Lymphoma	I	STING protein	SNX281
	NCT02727803	Personalized NK Cell Therapy in CBT	II		Allogeneic Natural killer Cell Line NK-92
	NCT05472558	Clinical Study of Cord Blood-Derived CAR-NK Cells Targeting CD19 in the Treatment of Refractory/Relapsed B-cell NHL	I	CD19	CAR-NK cells
	NCT04639739	Anti-CD19 CAR-NK Cell Therapy for Relapsed or Refractory B-cell NHL: a Multi-center, Uncontrolled Trial	I	CD19	CAR-NK cells
	NCT04796688	Safety and Efficacy of Universal Chimeric Antigen Receptor-modified AT19 Cells in Patients with CD19+ Relapsed/Refractory Hematological Malignancies: a Single-center, Open-label, Single-arm Clinical Study	I	CD19	CAR-NK cells
	NCT05379647	QN-019a as a Monotherapy and in Combination with Anti-CD20 Monoclonal Antibodies in Subjects with B-cell Malignancies	I	CD19	QN-019a, CAR-NK cells

*Abbreviations:* *Tfh* T follicular helper, *Treg* T regulatory, *TAMs* Tumor-associated macrophages, *MDSCs* Myeloid-derived suppressor cells, *CAFs* cancer-associated fibroblasts, *NK* Natural Killer, *ILCs* Innate lymphoid cells, *DLBCL* Diffuse Large B-cell Lymphoma, *MZL* Marginal Zone Lymphoma, *FL* Follicular Lymphoma, *iNHL* Indolent B-cell Non-Hodgkin’s Lymphoma, *HL* Hodgkin Lymphoma, *CLL* Chronic Lymphocytic Leukemia

**Table 3:** FDA-approved agents targeting the immune cells of the TME in B cell lymphomas

Agent	Drug Category	Indication	Approval Year	Trials
Pembrolizumab	CPI	Adult and pediatric patients with refractory PMBCL	2018	KEYNOTE-170 (NCT02576990)
Lenalidomide	IMIDs	Previously treated FL and MZL	2019	AUGMENT (NCT01938001); MAGNIFY (NCT01996865)
Lenalidomide	IMIDs	ASCT-ineligible R/R DLBCL patients	2020	L-MIND (NCT02399085); RE-MIND (NCT04150328)
Umbralisib	PI3Ki	R/R MZL with at least one prior anti-CD20-based regimen; R/R FL with at least 3 prior lines of systemic therapy	2021	UTX-TGR-205 (NCT02793583)
Zanubrutinib	BTKi	R/R MZL with at least one anti-CD20-based regimen	2021	BGB-3111-241 (NCT03846427); BGB-3111-AU-003 (NCT02343120)

*Abbreviations:* *CPI* checkpoint inhibitor, *IMIDs* immunomodulatory drugs, *PI3Ki* phosphoinositide 3-kinase inhibitor, *BTKi* Bruton Tyrosine Kinase inhibitor, *PMBCL* primary mediastinal large B cell lymphoma, *FL* Follicular Lymphoma, *MZL* Marginal Zone Lymphoma, *DLBCL* Diffuse Large B-cell Lymphoma, *ASCT* autologous stem cell transplant, *R/R* relapsed/refractory

### T follicular helper cells

**Fig. 1:**
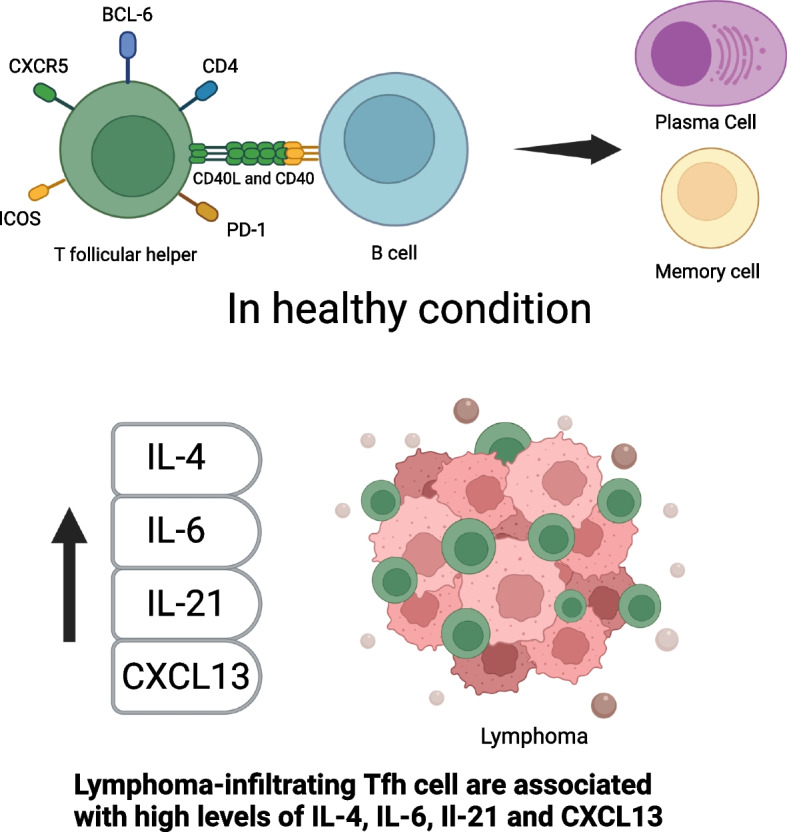
Role of T follicular helper (Tfh) cells in the normal germinal center and in lymphomagenesis

T follicular helper (Tfh) cells commonly reside inside the lymph nodes, tonsils, and spleen. They are defined by the expression of cell surface markers CD4, CXCR5, PD1, and ICOS, their master regulator being B cell lymphoma (BCL) 6 [15]. Tfh cells play a critical role in the formation and maintenance of GCs. Also, Tfh cells engage GC B cells to promote clonal selection and affinity maturation so that high-affinity B cells can be selected to exit the GC reaction and undergo terminal differentiation towards plasma cells or memory cells [15]. This mechanism is mediated through interaction between the co-stimulatory molecule CD40-ligand on the Tfh cells with CD40 on the B cells (Fig. 1) [15]. On the contrary, T follicular regulatory (Tfr) cells limit the output of the GC reaction counterbalancing Tfh function [1]. Of note, Tfh cells can convert to Tfr cells through FOXP3 activation in the late germinal center [16]. Several studies have shown an increased expression of Tfh CD4^+^PD1^+^ICOS^+^ cells [17] and/or CD4^+^CXCR5^+^Foxp3^-^ [18] cells in diagnostic samples of malignant lymphoid disease compared to healthy controls. The same expression decreased or returned to normal at the end of effective treatment, but it increased in progressive disease [17]. It is possible that Tfh cells may contribute to lymphoma B cell survival via production of sCD40L which activates NF-kB pathway and in turn up-regulates c-FLIP and Bcl-xL [19, 20]. Increased expression of lymphoma-infiltrating Tfh cells was associated with high levels of IL-6, IL-21 [21], IL-4 [22], and CXCL13 [9] (Fig. 1). Conversely blocking these cytokines resulted in reduced infiltration of Tfh cells [21]. Additionally, the crosstalk between lymphoma B cells and Tfh cells increases the release of CCL17 and CCL22, which induces the preferential migration of regulatory T cells (Treg) and IL-4 producing CD4^+^ T cells, stimulating more chemokine release thus creating an immune suppressive TME that promotes tumor survival and growth [23, 24]. Another study divided Tfh cells into Tfr-like subsets (CD4^+^CD25^+^CXCR5^+^) and Tfh CD25^-^ subset (CD4^+^CD25^-^CXCR5^+^) [25]. The difference between these two groups was associated with the higher expression of Blimp1, Foxp3, IL-10, TGF-β, and lower levels of IL-21 in Tfr-like CD25^+^ cells compared to Tfh CD25^-^ cells [25]. This discovery is intriguing as it demonstrates the plasticity of the immune response and implies the possibility to leverage this characteristic as a therapeutic tool. Novel insights on the role of Tfh cells in immune evasion can usher in the opportunity for unexplored therapeutic targets [26]. In particular, identification of genetic mutations, cell markers and cytokine/chemokine signaling that impact Tfh cell function will help in improving our knowledge of the causative events that induce and/or sustain tumor development and growth. Thus, targeting these regulators may be a new approach to interrupting T cell support of lymphoma cells, which may complement other therapeutic approaches.

### T regulatory cells

Treg cells are CD4^+^ T cells expressing high CD25 (IL-2Rα) and FoxP3, and low or not CD127 (IL-7R α) [27, 28]. They suppress immune response activation and promote tolerance towards self-antigens to prevent autoimmunity [29]. However, their function can also suppress tumor immunity leading to immune escape [30]. Nevertheless, the significance of tumor-infiltrating Treg cells remains elusive due to their heterogeneity and their expression of both co-inhibitory and co-stimulatory receptors [31]. Specifically, some studies have shown that Treg FOXP3^+^ cells display a tumor-protective effect [32, 33] in FL [34] and DLBCL [34, 35] by suppressing T-cell proliferation and IFN-γ production [31, 36], while others found that Treg cells co-expressing activating markers such as CTLA4 [37] and TIGIT [38] result in an enhanced suppressive property and are associated with poor prognosis [39]. It is possible that the prognostic impact of Treg cells is dependent on disease context, however more clarity is still needed. Therefore, in-depth phenotypic and functional characterization of Treg cells is mandatory to identify novel targets for therapy and in turn improve patient survival. These data suggest that targeting Treg cells could be beneficial due to their antitumor immunity, however, it might also lead to unwanted immune-mediated toxicities.

**Fig. 2:**
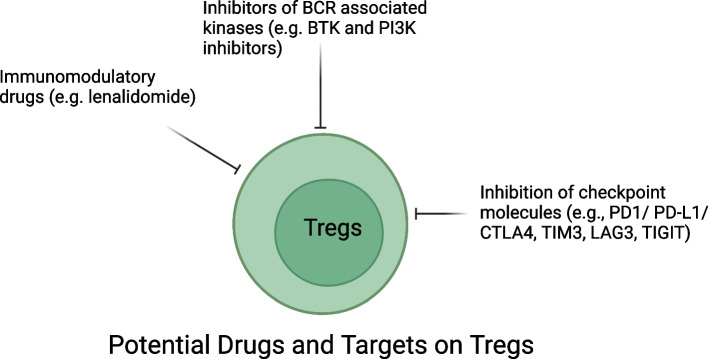
Drugs targeting T regulatory (Treg) cells

In the last decade several immunomodulatory drugs (IMiDs) (e.g. lenalidomide) and targeting agents against B cell receptor (BCR) or intracellular kinases (e.g. BTK inhibitors and PI3K inhibitors) have been approved for hematologic malignancies [40]. Beside the tumor-specific effect, these molecules can also impact the immune components of the microenvironment (Fig. 2). For example, lenalidomide modulates Treg cells decreasing their suppressive function [41–43] and results in an enhanced anti-lymphoma activity. Similarly, PI3K inhibitors decrease the suppressive effect of Treg cells while enhancing CD8 T cell function [44–46]. The most recent therapeutic strategies targeting T cells include inhibition of checkpoint molecules such as PD1/PD-L1 and CTLA4 [47] or adoptive transfer of genetically engineered T cells [48]. Additional recently discovered immune checkpoint molecules that represent emerging targets for therapy are TIM3, LAG3 and TIGIT [49]. Blocking the negative T cell regulator CTLA4 reactivates immune response against the tumor in immunogenic cancers [50]. CTLA4 inhibition decreased Treg cells also in B cell lymphoma with a positive association of CD45RA-Treg ratio in responders vs non responders, however the antitumoral effects were quite modest [51]. PD1/PD-L1 inhibition prevents T cell exhaustion [52] and blocks the suppressive Treg activity [53]. Interestingly, inhibition of one checkpoint leads to compensatory increase of others. For example, blocking PD1 results in increase of LAG3 and CTLA4 [54]. On the contrary, combined inhibition of PD1 and LAG3 increased CD8 T cell cytotoxicity and decreased Treg cells [55]. Nevertheless, combination of two checkpoint blockades has shown modest activity in relapsed/refractory (R/R) B cell lymphoma [51, 56]. Similar to LAG3, TIM3 results in negative regulation of T cell response, ultimately leading to T cell exhaustion [57, 58], while its inhibition reduces tumor growth especially in combination with PD-1 blockade, but again the overall anti-tumor effect is modest [59]. TIGIT is also a negative regulator of T cells that can prevent immune response against tumor [60, 61]. As such it has attracted scientific attention as a novel target for therapy [62] and its use is under experimental evaluation. Given the tremendous potential of immune therapy, optimal methods to modulate Treg cells are needed in the future to achieve a balance between antitumor immunity and autoimmunity.

### Effector CD8+ T cells

Naïve CD8^+^ T cells differentiate into cytotoxic effector CD8^+^ T cells when encountering a cognate antigen [63]. Once the antigen has been eliminated, they undergo apoptosis or differentiate into memory T cells [64]. However, CD8^+^ T cells may become exhausted in the face of persistent antigen stimulation in infections or autoimmunity [65]. In addition, during tumorigenesis cancer cells secrete inhibitory factors to generate an immune suppressive tumor environment, thus, despite their important role in eliminating tumor cells, CD8^+^ cytotoxic T cells often become exhausted and eventually fail to control tumor development and progression [65]. Anergic or exhausted CD8^+^ T cells are defined as CD8^+^ CD28^-^ CD57^+^ T cells with a reduced proliferation and cytotoxic effect (loss of IL-2, TNF-α, and IFN-γ production) [66]. Differential expression level of CD5 distinguishes different T cell activation and effector function, as CD5^high^ CD8^+^ T cells are more active and abundant in the TME compared to CD5^low^ CD8^+^ T cells. Since CD5 expression inversely correlates with PD1 expression, targeting CD5 may increase PD1 levels, which in turn would maximize the effect of anti-PD1 checkpoint blockade [67]. CD8^+^ T cells are also characterized by a sustained expression of inhibitory receptors such as PD1, CTLA4, and LAG3 [68]. Several studies have shown a favorable correlation between increased numbers of effector CD8^+^ T cells and good outcomes in FL [69, 70]. Specifically, increase of PD1^+^ CD8^+^ T cells associated with a favorable outcome in FL patients, while reduction of the same was observed in transformation [71]. By contrast, expression of LAG3 defines a subset of PD1^+^ CD8^+^ T cells which correlates with poor outcome in FL [72]. In line with these data, inhibition of LAG3 increases the proliferation and effector function of CD8^+^ T cells [73], suggesting that these immune checkpoint inhibitors can potentially augment antitumor immunity. Currently, there are several clinical trials investigating the efficacy of anti-LAG3 inhibitors alone or in combination with other immunotherapy in hematologic malignancies (NCT04566978, NCT05039658, NCT02061761, NCT05255601).

### Tumor-associated macrophages

**Fig. 3:**
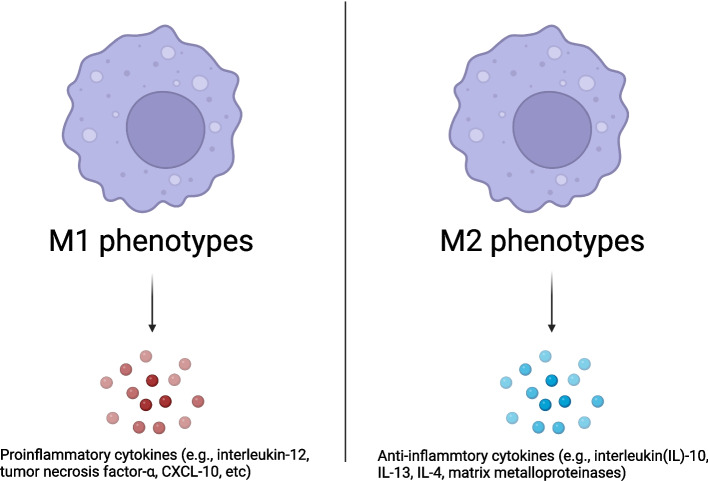
Macrophage polarization and specific cytokines release of M1 and M2 macrophages

Tumor-associated macrophages (TAMs) are one of the most critical immunosuppressive cell populations. TAMs suppress antitumor immunity and promote tumor progression by inhibiting the recruitment and activation of T cells via secreting cytokines, chemokines, and growth factors [74]. TAMs also serve as prominent metastasis promoters in the TME [75]. TAMs are classified into M1 and M2 phenotypes. In general, M1 macrophages are cytotoxic via secreting proinflammatory cytokines (e.g., IL-12, tumor necrosis factor-α, CXCL-10) and are considered anti-tumorigenic, while M2 macrophages are pro-tumorigenic via secreting anti-inflammatory cytokines (e.g., IL-10, IL-13, IL-4, matrix metalloproteinases) [75] (Fig. 3). A study by Taskinen et al. showed that high expression of CD68^+^ (M1 marker) TAMs was associated with adverse outcome in chemotherapy-treated FL patients (*P* = 0.026), but those patients had a favorable prognosis (progression free survival [PFS] was not reached, *p* = 0.006) and overall survival (p = 0.006) compared to the control group [76]. However, an increased number of TAMs, particularly CD68^+^ macrophages, was correlated with an increased likelihood of relapse after autologous hematopoietic stem-cell transplantation (*P* = 0.008) and shortened PFS (*p* = 0.03) in patients with classic Hodgkin’s lymphoma (HL) [77]. Along the same lines, elevated numbers of infiltrating CD163^+^ M2 macrophages were associated with increased angiogenic sprouting and poor prognosis in FL [78] and DLBCL [79]. Therefore, TAMs may exert either antitumor or protumor functions in different tumor types [80].

Many clinical approaches targeting TAMs are still under investigation. Targeting the colony-stimulating factor-1 receptor (CSF1R) signaling pathway, which is essential for the recruitment, differentiation, and survival of TAMs, leads to their decrease in number and in immunosuppressive functions [81]. Targeting CSF1R caused abrogation of CD163^+^ TAMs in mantle cell lymphoma (MCL), irrespective of the sensitivity to BTK inhibitors [82]. PLX3397 (pexidartinib), a CSF1R inhibitor, significantly reduced the viability of M2 macrophages, but it did not affect M1 macrophages in FL [83]. Also, inhibition of CSF1-CSF1R axis improved the efficacy of other immunotherapies, such as PD-1 or CTLA-4 blockades [84]. Another promising target is CD47 which is overexpressed in several B cell lymphomas, including DLBCL, FL and MCL [85]. The interaction between CD47 and SIRPα prevents cancer cells from being phagocytosed by macrophages and dendritic cells [86]. Chao et al. reported that anti-CD47 antibody reduced lymphoma burden, and the combination with rituximab had a synergist effect on promoting phagocytosis of lymphoma cells [85]. Notably, anti-CD47 antibodies robustly inhibited the dissemination of disease to secondary sites [87]. This correlated with a benefit in prognosis as extranodal lymphomas generally associate with a reduced response to therapy and a worse prognosis. CCL2/CCR2 is another essential signaling axis implicated in activating and mobilizing TAMs from the bone marrow to the site of inflammation in the TME [88]. Targeting CCL2-CCR2 might be a feasible immune intervention for lymphoma treatment. A study showed that CREBBP/EP300 mutation in DLBCL patients had higher CCL2 expression, and tumor progression was induced by TAMs throughout the FBXW7-NOTCH-CCL2/CSF1 axis [88]. Accordingly, CCR2 antagonist decreased tumor growth and dissemination of DLBCL cells, and increased survival in xenograft models [89]. Another study showed that the combination of CCR2 and immune checkpoint inhibitors reduces tumor growth in cutaneous T-cell lymphomas [90]. Lastly, microRNAs (miRNA) are secreted from tumor cells and could induce the recruitment and reprogramming of TAMs [91]. Recent studies have shown that overexpression of specific miRNAs (e.g., miR-33, miR-130, and miR-155) decreases tumor progression by shifting TAM from M2 to M1 phenotype [92, 93].

### Myeloid-derived suppressor cells

**Fig. 4:**
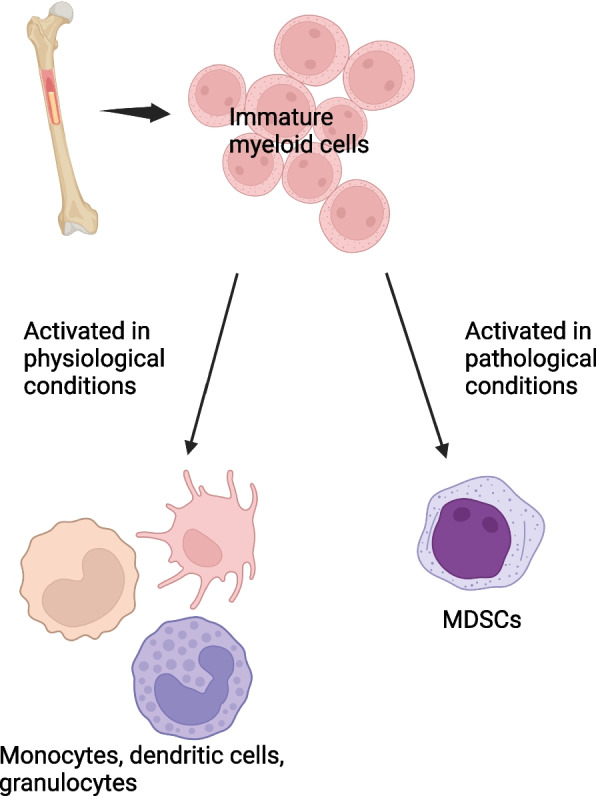
Myeloid differentiation in physiologic and pathologic conditions

Myeloid-derived suppressor cells (MDSCs) are a heterogeneous group of immature myeloid cells (IMC) which is pathologically activated in many conditions, including autoimmunity, infectious diseases, obesity, and pregnancy [94]. In physiological conditions, IMCs differentiate into mature monocytes, dendritic cells, and granulocytes, however the differentiation and maturation of IMCs are blocked in a pathological environment, which leads to the expansion of MDSCs (Fig. 4) [95]. MDSCs are further divided into two major subsets: polymorphonuclear (PMN)-MDSCs and monocytic (M)-MDSCs. They can be differentiated from their normal counterparts by high arginase-1 (Arg-1) and nitric oxide synthase-2 (NOS-2) expression, and high and persistent level of reactive oxygen species (ROS) [94]. Also, PMN-MDSCs can be distinguished from neutrophils by their unique genomic profile [94], while M-MDSCs are different from TAMs based on their phenotype characterized by increased expression of F4/80 and M-CSF receptor, low expression of IRF8, low to intermediate expression of Ly6C and low or undetectable expression of S100A9 protein [94, 96]. MDSCs were shown to be higher at the time of diagnosis in Hodgkin and Non-Hodgkin lymphoma patients, especially in those with aggressive disease, compared to healthy control [97, 98]. Upregulated expression of MDSCs-related genes (e.g. ARG1, S100A12, and S100A8) was associated with inferior event-free survival compared to patients with low expression of these genes [99, 100]. Endoplasmic reticulum stress is the main regulator of the activation and suppressive function of MDSCs by promoting the expression of Arg-1 and NOS-2 [95]. Also, exosomes released by cancer cells accelerate the activation, expansion, and immunosuppression of MDSCs by transporting functional substances, such as miRNA, TGF- β, and PGE2 [95, 101].

Promising therapeutic strategies are reducing MDSCs accumulation in the TME as well as inducing functional repolarization of these cells. However, a complete deletion of myeloid cells would not be feasible as it may cause severe adverse effects, such as bacterial infections. An example of such a strategy is phosphodiesterase-5 inhibitors (e.g. sildenafil) which reduce the immunosuppressive effect of MDSCs and enhance intratumoral T cell infiltration and activation through downregulation of Arg-1 and NOS-2 [102]. Antagomir, an antagonist of miR-30, showed to reduce MDSCs in B-cell lymphoma [103]. Histamine dihydrochloride (HDC) with IL-2 reduced MDSCs, but this anti-tumor mechanism is insufficiently understood [104]. The PI3Kδ/γ inhibitor RP6530 led to a significant inhibition of MDSCs and repolarized TAMs from M2 to M1-like phenotype in Hodgkin lymphoma (HL) in vitro and in vivo [105]. In the future, targeting MDSCs may be a crucial point to improve the efficacy of CAR-T cell therapy since it has been shown that MDSCs could inhibit CAR-T cell activation [106, 107].

### Cancer-associated fibroblasts

**Fig. 5:**
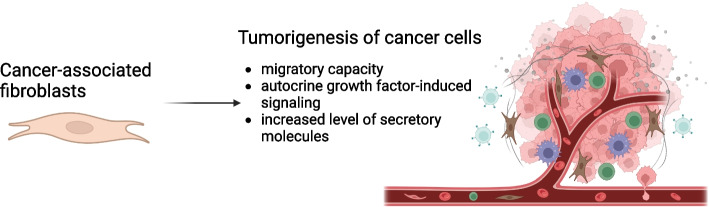
Functions of cancer-associated fibroblasts (CAFs)

Fibroblasts are resting mesenchymal cells in the connective tissue, which become activated during wound healing by growth factors, such as TGF-β, platelet derived growth factor (PDGF) and IL-6 [108]. Once activated, fibroblasts generate cytokines and chemokines, recruit immune cells, and synthesize an extracellular matrix (ECM). However, normal activated fibroblasts are different from cancer-associated fibroblasts (CAFs). CAFs exhibit enhanced migratory capacity, autocrine growth factor-induced signaling and increased levels of secretory molecules that enhance tumorigenesis (Fig. 5) [108]. This process might be a consequence of epigenetic changes promoting CAFs activation. Among the different molecular regulators released by CAFs, the CAF-derived stromal cell-derived factor 1 promotes tumor growth by inducing angiogenesis via the recruitment of endothelial progenitor cells into tumors [109]. CAFs also produce abundant VEGF, PDGFC, FGF2, osteopontin and secreted frizzled-related protein 2 to exacerbate the angiogenesis of neoplastic tissues [110]. Heat shock factor 1 (HSF1) may cause HSF1-driven pro-tumorigenic program in cancer cells [111]. Yes-associated protein 1 enhances stiffening of ECM, angiogenesis, and cancer cell invasion [112]. In general, CAFs promote an immune suppressive TME. The cytokines or chemokines secreted by CAFs may have direct or indirect implications for tumor immunity [110]. It is uncertain if CAFs are associated with immunosuppressive populations of B cells due to poorly defined markers for such cells [113]. Production of IL-4, IL-6, and IL-8 may induce immunosuppressive myeloid cell differentiation, while CXCL14 affects macrophages recruitment to the tumor. Additionally, CAFs modulate immunity through their acquisition of adhesion molecules (e.g., ICAM1), which serve as a docking platform for the immune cells [114].

Lymphoma B cells can trigger mesenchymal stem cells (MSCs) differentiation into fibroblast reticular cells. Pandey et al. reported that stromal cells of FL-infiltrating lymph nodes and bone marrow overexpressed CXCL12, while IL-4-high FL-Tfh cells triggered CXCL12 upregulation [115], which further promotes FL B cell activation, migration and adhesion [115]. IL-8 promotes neutrophil survival, causing activation of stromal cells and promotion of malignant B-cell survival [116]. CCL2 is overexpressed by MSCs from FL bone marrow in comparison with those from healthy age-matched donors (HD-MSCs), and it is up-regulated in HD-MSCs after coculture with malignant B cells [117]. DLBCL stromal-1 gene signature is enriched in CAFs and its expression is inversely associated with DLBCL tumor stage. Thus, CAFs are hypothesized to aid in trapping malignant B cells in the lymph node preventing their spread to new anatomical locations. Among all the gene regulators, TGF-β is the main upstream regulator of the DLBCL stromal-1 gene signature [118]. TGF-β has been shown to cause apoptosis in mouse models of B cell lymphoma [119]. Although TGF-β could promote an immunosuppressive environment, it is also a potent negative regulator of B-cell survival, proliferation, activation, and differentiation [120].

Targeting CAFs could be a challenging task due to the lack of specific cell surface markers causing difficulty to precisely target CAFs without damaging the normal tissue. However, there are a few general approaches targeting CAFs: 1) targeting the chemokine and growth factor pathways to inhibit the activation of CAFs, 2) normalization of CAFs via all-transretinoic acid or calcipotriol, 3) depletion of CAFs by transgenic technologies or immunotherapies, 4) targeting CAF-derived ECM proteins and associated signaling to induce stromal depletion, 5) cellular therapies (such as oncolytic adenoviruses, TNF-related apoptosis-inducing ligand or type I interferon) [110].

### Tumor-infiltrating natural killer cells

**Fig. 6:**
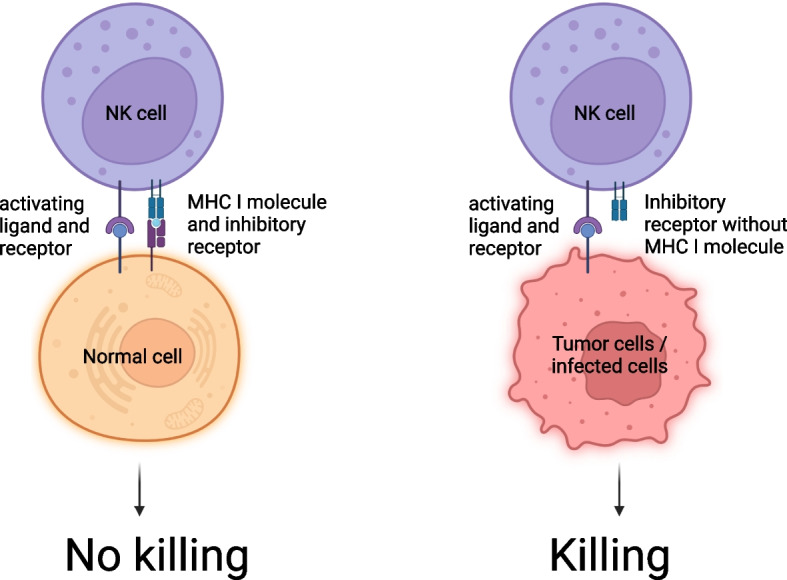
Role of natural killer (NK) cells in physiologic and pathologic conditions

Natural killer (NK) cells are innate cytotoxic lymphocytes of the immune system, contributing to the prevention of infection and tumor growth [121]. NK cells can be divided into two subtypes: CD56^dim^ CD16^+^ NK cells (a mature cytotoxic population) and CD56^bright^ CD16^-^ NK cells (an immature and mostly immunomodulatory population) [121]. For both populations the most important cell surface inhibitory receptors are i) the members’ killer cell immunoglobulin-like receptor (KIR) family and ii) the CD94/NKG2A heterodimer [122, 123]. In physiologic conditions, normal cells are spared by the NK cells due to the recognition of MHC Class I engaged with KIRs. By contrast, lack of “self-recognition” signals to the NK cells to attack abnormal cells, such as tumor cells which present downregulated antigen presentation molecules as immune evasion strategy (Fig. 6) [124].

The role of NK cells in tumor immunosurveillance is well established [125, 126]. Importantly, NK cells seem to prevent development of tumors including B cell lymphoma [127, 128]. Recent evidence has shown that tumor infiltrating NK cells unleashed cytotoxic T cells, ultimately resulting in tumor eradication [129]. In line with the role of NK cells in suppressing malignancies, several studies have demonstrated a survival advantage of tumor infiltration by NK cells [129–133]. Even though a direct correlation may be less clear due to the frequent co-expression of T cells, these studies support a critical role of NK cells in promoting antitumor immune response. Tumor immune escape includes mechanisms that prevent NK cell activation or recruitment. For examples, suppressive cytokines (e.g. TGF- *β*) [134, 135] and prostaglandin [136, 137] clearly suppress NK cell activation. TGF- *β* also induces differentiation of Treg cells, which in turn suppress NK cells [138, 139]. Additional escape mechanisms include engagement of inhibitory receptors. Besides expressing NK-cell inhibitory receptors, NK cells also express other immune checkpoint molecules (e.g., PD1, TIM3, TIGIT, SIRP *α*) [140–144]. For example, increased expression of PD1 on NK cells was observed in several tumors [145–148], including HL and DLBCL [148]. By contrast, the inhibitory ligand PDL1 was found on tumor cells and macrophages, thus favoring the PD1/PDL1 interaction which limits the anti-tumor effect of NK cells. Recent studies have shown that PD1 blockades disrupt the suppressive PD1/PDL1 axis, reactivating NK cells with clinical implication [148]. Blockade of other immune checkpoint molecules has also shown encouraging potential for NK cell-based immunotherapy [124]. TIGIT was associated with NK cell exhaustion. On the contrary, TIGIT blockade antibodies restored anti-tumor activity [149]. Monalizumab, a humanized antibody against NKG2A, unleashes NK and T cells, thus promoting an enhanced tumor immunity [150]. STING agonists, such as cyclic dinucleotides, enhance NK cell fitness and anti-tumor effect [130, 151]. Another approach to amplify NK cell function against tumor is using “NK cell engagers”: bi- or tri-specific antibodies that bind NK and tumor cells [152, 153]. Furthermore, FDA has recently approved the first NK cell-based immunotherapy, NK-92, for clinical testing [154, 155]. Of note, NK cells provide a safer chimeric antigen receptor (CAR)-engineering platform compared to T cells [156]. Additionally, since they lack most of the KIRs, CAR-NK cells are less likely to become exhausted [157]. Several ongoing efforts have attempted to further potentiate and prolong NK-CAR potency by combining checkpoint inhibitor, cytokines and co-stimulatory signaling [157]. However, this promising off-the-shelf approach needs additional improvements to maximize its therapeutic efficacy.

### Innate lymphoid cells

Innate lymphoid cells (ILCs) belong to the adaptive immune system and have a similar phenotype and function of T cells but differ from them for the lack of antigen receptors and clonal selection and expansion after stimulation [158]. ILCs are relatively rare (≤ 1% lymphocytes in mucosal tissues) [159] and can be distinguished in three main subsets: 1) type 1 ILCs include ILC1s and conventional NK cells [160, 161], express Tbx21, produce IFN-γ, and contribute to anti-viral and Th1 immunity [162]; 2) type 2 ILCs express Gata3, ROR *α*, TCF1 and Notch [163, 164], produce Th2 cell-associated cytokines (IL-4, IL-5, IL-9 and IL-13), and contribute to respond to Helminths infections and allergic diseases [165]; 3) type 3 ILCs express ROR *γ* t, present a different expression of T-bet [161, 166–168], produce IL-17A and IL-22, and participate in the homeostasis and mucosal defense and preservation of memory CD4 T cells [164, 169]. Notably, ILCs have a remarkable plasticity that allows them to acquire features of another ILCs subtype as required by changes in the TME. For examples, NK cells can switch to ILC1-like cells upon increase of TGF- β [135]. The existence of a continuous conversion from NK cells to ILC1s and vice versa is also plausible [170, 171]. Similarly, IL-12 has been shown to induce differentiation of ILC2s into ILC1 [172, 173] and ILC3s into ILC1s [173, 174]. ILCs also regulate tumor surveillance through a dynamic crosstalk with different immune components of the TME. Among ILCs, NK cells are the most active population as previously described. ILC2s can suppress immune response against tumor through IL-13-mediated enhancement of MDSCs expansion [174], alternatively they favor anti-tumor immunity through IL-5-mediated cooperation with DCs [175, 176]. ILC2s may potentiate the suppressive function of Treg through release of the growth factor AREG [177], or limit T cell activation through production of Arg1 [178]. ILC3s favor chronic inflammation, which in turn may promote tumor initiation [179, 180]. A group of ILC3s produce IL-17 and IL-22 [181, 182], which have been associated with poor prognosis in cancer patients [183, 184]. Collectively, these studies support the interplay between ILCs and the immune cells of the TME, which influence both innate and adaptive immune response against tumor. Future studies may be directed to investigating strategy blocking ILCs-myeloid or ILCs-Treg axes as a promising therapeutic strategy.

### Lymphomas of the immune-privileged sites

The lymphomas of the immune-privileged sites include those arising from the central nervous system (PCNSL) and testes (PTL) [185]. Unlike other lymphomas, PCNSL and PTL are invisible to the immune system and have a suppression of anti-tumor T-cell response. Typically, they are localized diseases at presentation, even though they may be disseminated within the compartment (CNS-CNS, testis-testis) and between the compartments (CNS to testis) but rarely systemically, and have a poor prognosis [186, 187]. Constitutive activation of NF-kB via BCR (e.g. *CD79B* mutation) and toll-like receptor (e.g. *MYD88 L265P* mutation) is the canonical oncogenic pathway [188–190]. They share genetic features with classical ABC-DLBCL as well as with the recently defined molecular clusters MCD and C5 [4, 5]. However, the precise relationship between these classes remains to be elucidated. They present a high prevalence of genetic mutations causing loss of MHC class I and II expression [189, 191, 192]. Additionally, structural alterations at 9p24.1, which is the PD-L1 and PD-L2 locus, increase the abundance of transcriptional and translational expression of PD-L1 and PD-L2, further reinforcing immune evasion [189]. The predominant immune components of TME in these diseases are CD8^+^ cytotoxic T cells with a direct correlation between their number and outcome. Macrophages are also frequently identified, being an increased M1/M2 ratio associated with a better survival. Of note, PD1 and TIM3 appear to be concomitantly upregulated in CD8^+^ cytotoxic T cells and M2 macrophages with prognostic implications [193, 194]. However, further investigation is required to uncover the immune landscape of these diseases. The specific features of the lymphomas of the immune-privileged sites impact on treatment option. Especially, NF-kB/BTK inhibition has shown promise, with ibrutinib-based therapy being at the forefront of clinical investigation [195–197]. Additionally, checkpoint inhibition (e.g. nivolumab/pembrolizumab) has had an emerging role in the therapeutic armory [198].

## Conclusion

The crosstalk between malignant B cells and immune cells in the lymphoma TME is highly complicated and might be affected by often interconnected intrinsic and/or extrinsic mechanisms which ultimately can lead to immune escape. This notion suggests the need to adopt a more comprehensive therapeutic strategy that does not limit its focus to tumor cells but that considers a global approach including the TME. Targeting the TME has long been considered a promising strategy, but much more work is needed to identify novel prognostic and predictive targets. Stratification of the patients for precision medicine as well as monitoring of immune response remain unmet clinical needs. Several advancements have been made towards this direction, such as the recent development of liquid biopsy that monitors circulating tumor DNA and immune components [199] or immune-imaging tools [200–202] to assess the efficacy of immunotherapy. The horizon of B cell lymphoma allows for a glimpse of a therapeutic strategy that considers the tumor in its whole, and maybe such an approach might be able to overcome the current clinical hurdles and rescue the still high therapeutic failures.

## Data Availability

Not applicable.

## Abbreviations

GC: Germinal center
GEP: Gene expression profiling
DLBCL: Diffuse large B cell lymphoma
COO: Cell of origin
ABC: Activated B cell
GCB: Germinal center B cell
WES: Whole exome sequencing
FL: Follicular lymphoma
Tfh: T follicular helper
BCL6: B cell lymphoma 6
Tfr: T follicular regulatory
IMiDs: Immunomodulatory drugs
TAMs: Tumor-associated macrophages
PFS: Progression free survival
CSF1R: Colony-stimulating factor-1 receptor
miRNA: microRNAs
MDSCs: Myeloid-derived suppressor cells
IMC: Immature myeloid cells
PMN: Polymorphonuclear
Arg-1: Arginase-1
NOS-2: Nitric oxide synthase-2
PDGF: Platelet derived growth factor
ECM: Extracellular matrix
CAFs: Cancer-associated fibroblasts
HSF1: Heat shock factor 1
HD-MSCs: Healthy age-matched donors
HL: Hodgkin lymphoma
NK: Natural killer
CAR: Chimeric antigen receptor
ILC: Innate lymphoid cells
PCNSL: Primary central nervous system lymphoma
PTL: Primary testis lymphoma
BCR: B cell receptor
